# 放疗技术与放射性肺损伤：高剂量小体积还是低剂量大体积？

**DOI:** 10.3779/j.issn.1009-3419.2015.12.07

**Published:** 2015-12-20

**Authors:** 冰琪 喻, 谨 王, 裕金 徐, 峰 苏, 国平 单, 明 陈

**Affiliations:** 1 310013 杭州，浙江省肿瘤医院放疗科/浙江省放射治疗重点实验室 Department of Radiation Oncology, Zhejiang Cancer Hospital, Zhejiang Key Laboratory of Radiation Oncology, Hangzhou 310022, China; 2 310013 杭州，浙江省肿瘤医院物理室 Department of Physics, Zhejiang Cancer Hospital, Zhejiang Key Laboratory of Radiation Oncology, Hangzhou 310022, China

**Keywords:** 肺肿瘤, 放射性肺炎, 三维适形放疗, 调强适形放疗, Lung neoplasms, Radiation pneumonitis, Three-dimensional conformal radiation therapy, Intensity modulated radiation therapy, Hybrid intensity modulated radiation therapy

## Abstract

放射治疗是肺癌的主要治疗手段之一，目前使用的主流技术是三维适形放疗（three-dimensional conformal radiation therapy, 3D-CRT）和调强适形放疗（intensity modulated radiation therapy, IMRT），两者各具特点。本文综述近年来两种放疗技术治疗肺癌的文献，重点讨论放射剂量在肺内的分布与放射性肺炎的关系，即高剂量分布在较小的肺体积与低剂量分布在较大的肺体积，两者哪种更易引发放射性肺炎（radiation pneumonitis, RP）。

## Abstract (en)

Radiotherapy is one of the main treatment for patients with lung cancer. Three-dimensional conformal radiation therapy (3D-CRT) and intensity modulated radiation therapy (IMRT) are widely used to deliver radiation. Here, we focus on the correlations between dose distribution in lung and radiation pneumonitis according to the analysis about radiotherapy for lung cancer: A lot to a little or a little to a lot, which is the main cause of radiation pneumonitis?

## 1 引言

放射治疗简称放疗，是肺癌的主要治疗方式之一^[1]^，三维适形放疗（three-dimensional conformal radiation therapy, 3D-CRT）和调强适形放疗（intensity modulated radiation therapy, IMRT）成为主要放疗技术，其中IMRT技术的使用正呈逐年上升趋势^[2, 3]^。由于3D-CRT技术及IMRT技术各具特点，这两种技术的差异是否会对临床治疗肺癌造成不同的影响？

## 2 定义及简介

3D-CRT是指通过一系列不同权重，不同射野形状和大小，从不同的方位向靶区进行分散照射的多个射线束照射技术，并采用与病灶形状一致的适形挡铅，它使得高剂量区在三维空间与靶区形状一致，同时降低靶区周边正常组织的剂量。

IMRT通过“子野”技术等调整放射野内射线强度，改善高剂量区域剂量分布与靶区形状的适合度，在一定程度上可避开正常组织，有效降低正常组织剂量且保持较好的适形度，使得靶区较3D-CRT更加精确。但靶区越精确，对摆位误差、评测器官运动、组织形变调整等要求越高。否则，随着呼吸运动、心脏跳动、器官组织放疗中的形状变化等，靶区可能移动到照射野外，靶区边缘剂量有可能达不到临床要求，造成局部控制率减低、肿瘤复发几率升高等^[4]^。IMRT使用许多子野调节剂量强度，对于肺癌、肝癌等移动靶区，子野实际剂量与计划剂量存在差异，是否影响疗效？IMRT危及器官低剂量区明显增加，其临床意义如何？

如果把肺作为一个并联器官，那么理论上就存在一个照射的体积阈值，小于这个阈值就不会发生功能性损害，超过这个阈值，将会产生一定程度的损伤，且随着照射剂量增加，功能性损害的严重程度增加。最初，3D-CRT被认为是“高剂量照射小体积”，IMRT是“低剂量照射大体积”，因此理论上后者所致放射性肺损伤更重。另外，照射时间也是评价放射性损伤的重要因素。Christian等^[5]^的研究中提到，进行5野IMRT技术放疗平均需要10 min，3野3D-CRT技术放疗只需要3 min。随着时间的延长，在呼吸运动等的影响下，正常组织的照射剂量无形中也会增加。结合这种假设，被认为是“低剂量照射大体积”的IMRT可能增加RP的发生率。但事实似乎并非如此。我们将从以下几个方面对两者进行比较。

## 3 疗效及损伤对比

### 3.1 3D-CRT和IMRT疗效对比

Harris等^[3]^、Techun等^[6]^和Chen等^[7]^对SEER数据库中行3D-CRT和IMRT的Ⅲ期非小细胞肺癌（non-small cell lung cancer, NSCLC）患者进行分析，并未发现两组总生存（overall survival, OS）有差异。Liao^[8]^在1999年-2006年对496例NSCLC患者进行同期放化疗，313例行CT结合3D-CRT（CT/3D-CRT），91例行4D-CT结合IMRT（4D-CT/IMRT），两组中位剂量均为63 Gy。在OS方面，4D-CT/IMRT组优于CT/3D-CRT组（*P*=0.037, HR=0.64, 95%CI: 0.41-0.98）。在局部无进展生存（loco-regional progression-free survival, LPFS）和远处无转移生存（distant metastasis-free survival, DMFS）上，两组无差异。说明在4D-CT引导下，IMRT技术的射线随肿瘤运动而改变，更能保证靶区剂量达到临床要求，延长OS，且不降低局部控制率（loco-regional control rate, LCR）。

### 3.2 3D-CRT和IMRT放射性肺损伤对比（表 1）

**1:** 3D-CRT和IMRT治疗中放射性肺炎发生率的比较 Comparision of radiation pneumonitis in patients treated with 3D-CRT and IMRT

Study	Treatment delivery	Radiation pneumonitis	*P* value
Shirvani *et al*^[2]^	3DCRT *vs* IMRT	37.5% *vs* 38.9%	0.220
Sue *et al*^[9]^	CT/3D-CRT *vs* 4DCT/IMRT^*^	the 6^th^ month 22% *vs* 8%	0.002
		the 12^th^ month 32% *vs* 8%	
	Subgrup^#^	the 6^th^ month 22% *vs* 4%	0.001
		the 12^th^ month 32% *vs* 4%	
Liao *et al*^[8]^	CT/3D-CRT *vs* 4DCT/IMRT	CT/3D-CRT > 4DCT/IMRT	0.017
3D-CRT: three-dimensional conformal radiation therapy; IMRT: intensity modulated radiation therapy; CT: computed tomography; 4DCT: four dimensional computed tomography; ^*^: the median gross tumor volume for 3D-CRT and IMRT were 142 and 194 mL, respectively. ^#^: amifostine was used when large amount of lung receiving 20 Gy.			

Shirvani等^[2]^对SEER数据库中3, 729例3D-CRT技术放疗患者和257例IMRT技术放疗患者进行放射性肺损伤的研究，两者放射性肺炎发生并无差异（*P*=0.220）。Yom等^[9]^的单中心研究中，入组2002年-2005年290例Ⅲ期NSCLC患者，这些患者皆采用根治性同步放化疗，68例采用4D-CT引导下IMRT技术放疗，222例用3D-CRT技术放疗。IMRT组中位肿瘤靶区（gross tumor volume, GTV）体积为194 mL，3D-CRT组中位GTV体积为142 mL（*P*=0.002）。IMRT组剂量无法达到临床要求的患者，放疗前予阿米福汀保护正常组织。结果显示在放疗开始后第6个月、12个月≥3级治疗相关放射性肺炎（treatment-related pneumonitis, TRP）的发生率上，IMRT组分别为8%和8%，3D-CRT组分别为22%和32%（*P*=0.002）。将IMRT组给予阿米福汀的患者剔除，为新的IMRT亚组，放疗开始后第6个月、12个月≥3级TRP的发生率均为4%，仍低于3D-CRT组（*P*=0.001）。Liao等^[8]^的研究中，CT/3D-CRT组V20（percentage volume of total lung exceeding 20 Gy）高于4D-CT/IMRT组（37% *vs* 34.4%），V5低于4D-CT/IMRT组（56.9% *vs* 64.5%）。结果CT/3D-CRT组≥3级RP的发生多于4D-CT/IMRT组（*P*=0.017）。说明IMRT未增加RP的发生，反而4D-CT/IMRT技术放疗提高了靶区的准确性，更能保护正常组织，减少RP的发生。

从现有研究来看，“小剂量照射大体积”并没有增加RP的发生，我们认为，可能原因有三点：首先，放疗所致肺发生功能性损害的体积阈值较高，具体阈值有待进一步研究。Claude等^[10]^的研究发现平均肺剂量（mean lung dose, MLD）、V20及V30与≥2级RP有关，我们之前研究^[11]^表明，急性放射性肺炎与剂量学参数MLD、V20、V30、V40、V50及正常组织并发症概率（normal tissue complication probability, NTCP）相关，而这些剂量学参数彼此存在很强的相关性，特别是在MLD、V20、V30之间; 在同步放化疗模式下，控制重度急性放射性肺损伤（severe acute radiation pneumonitis, SARP）发生率≤5%时，各剂量参数的临界值分别为：MLD≤16.77 Gy，V20≤34.15%，V30≤23.62%，V40≤18.57%，V50≤13.02%，MLD评估RP发生敏感性为78.0%，V20评估RP发生的特异性为82%。应用多个控制剂量学预测SARP可提高敏感性和特异性：可在MLD、V20、V30值中选一个或两个，V40、V50中的选一个。其次，照射相同体积的肿瘤，通过调整野的数目，3D-CRT可以实现“小剂量照射大体积”，IMRT也可以“大剂量照射小体积”。在不改变整个器官总的照射剂量前提下，正常组织的受照体积会随着照射野数目增加而增大^[12]^。此外，旁观者效应的存在使得损伤肺组织周边的未受照射的组织出现相同的病理改变，从而使损伤效应更明显^[13]^。靶区外未被照射的肺组织也表现出RP，发生RP的体积要大于靶区体积，即“大剂量”所照射的未必就是“小体积”。

## 4 联合化疗对放射性肺损伤的影响

同步放化疗或序贯放化疗已成为大多数局部晚期实体瘤的标准治疗手段，不同的化疗方式与不同的放疗技术联合可能造成不同的结果。Vogelius的^[14]^模型研究中，将化疗影响换算成放疗剂量，提出了化疗等效放疗剂量（chemotherapy equivalent radiation dose, CERD）的概念。随CERD增加，IMRT技术致肺损伤毒性作用较3D-CRT技术明显增加。Adkison^[15]^的研究发现，单纯放疗与诱导化疗+序贯放疗在1级、2级RP的发生率相似，放疗+巩固化疗却增加1级、2级RP的发生（*P*=0.018）。也就是说，在进行巩固化疗的患者中，IMRT技术致低级别RP发生率要高于非巩固化疗患者，此时，IMRT技术的低剂量大体积优势并不明显。可能与放疗之后进行的化疗增加损伤肺组织对放射性损伤的“回忆效应”^[16]^有关，“回忆效应”是指巩固化疗所致的组织反应，常发生在原接受放射治疗而未发生放射性损伤的组织或器官中。

## 5 基本状况对放射性肺损伤的影响

患者基础状况对RP的发生也有一定的影响。在Liao等^[8]^的研究中，经常吸烟患者≥3 RP的发生率明显低于从不/偶尔吸烟患者（*P*=0.003），类似的结果在其他研究^[17]^中也存在。Kimura等^[18]^对92例肺癌患者行累及野或加速超分割放疗，发现肺气肿越严重，患者RP发生率越低; 而接受SBRT治疗的患者中^[19]^，肺气肿程度与RP发生率呈正相关，即“小剂量照射大体积”时，肺气肿程度与RP发生率呈正相关，“大剂量照射小体积”时，肺气肿程度与RP发生率呈负相关。究其原因，我们考虑有：一方面，RP主要发生在正常肺组织，患者肺气肿越严重，正常肺组织体积越小，RP发生率越低; 另一方面，如前所述，V20可用于评估RP的发生，而在SBRT为代表的“小剂量照射大体积”中，V20较“大剂量照射小体积”时低，RP发生率较低。

## 6 新放疗技术对放射性肺损伤的影响

### 6.1 组合调强放疗技术可能聚集二者优点？

结合3D-CRT及IMRT技术特点，学者提出组合调强放疗技术（hybrid intensity modulated radiation therapy, h-IMRT）^[20]^。h-IMRT由3D-CRT及IMRT共同组成，利用人工干预的方式，根据肿瘤的形状及位置，给予2个-3个主野，再用IMRT技术补充，使剂量均匀分布于靶区，提高适形度。靶区不同，IMRT给予的剂量权重从0%-30%不等。结果发现，在V5、V20上，h-IMRT较3D-CRT及IMRT均有优势，且照射时间较IMRT明显缩短。

Verbakel等^[21]^对18例接受胸部h-IMRT放疗的患者进行剂量学研究，每个患者都做h-IMRT、3D-CRT、4野-5野IMRT及9野IMRT计划。结果：在V5、V13、V30上，h-IMRT组优于9野IMRT组（*P*=0.000, 5、*P*=0.000, 2、*P*=0.003），h-IMRT组与3D-CRT组相比，V5相似（*P* > 0.096），在健侧肺V13上，h-IMRT组较9野IMRT组野表现出优势（*P*=0.002, 1），说明h-IMRT技术放疗不仅能降低正常肺组织剂量，也能有效缩小受照体积，实现“低剂量照射小体积”，从而减轻肺损伤。但是该研究存在一个明显的缺陷，靶区剂量只限制了热点，靶区适形度较差，更为重要的是，危及器官只设置了肺和脊髓耐量，没有限制心脏和食管，使得纵隔器官受到高剂量照射，而心脏的放射损伤受到越来越多的关注，我们的病例研究表明一旦设置了靶区适形度和心脏食管限量，h-IMRT控制肺组织受量的优势即不再存在（图 1）。

**1:**
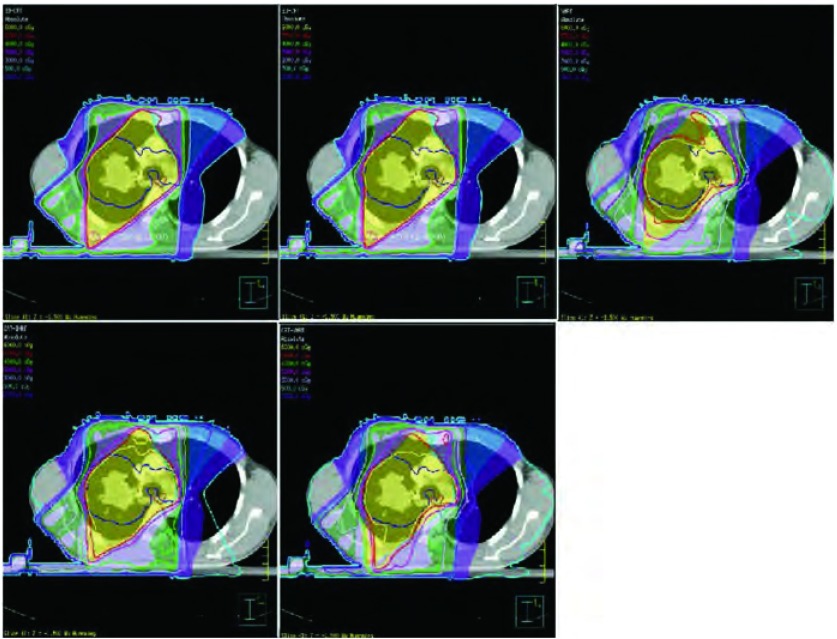
某患者肺癌平衡参数控制下的剂量参数图（从左到右分别为三维适形、调强适形、容积调强、组合调强和组合容积调强） Axial dose distribution for 5 radiotherapies(3D-CRT, full-IMRT, full-RapidArc, hybrid-IMRT and hybrid-RapidArc) for 1 special lung cancer patient under the dose limitation of lung, spinal cord, heart and esophageal

### 6.2 螺旋断层放射治疗更能保护正常组织？

随着螺旋断层放射治疗（helical tomotherapy, HT）的兴起，靶区剂量将更精确，正常组织可能受到更好的保护。HT集IMRT、图像引导放疗（image-guided radiotherapy, IGRT）、剂量引导放疗（dose-guided radiotherapy, DGRT）为一体，放疗同时进行在线剂量验证、位置验证。3D-CRT、IMRT和HT三种放疗技术在食管癌放疗的比较中^[22]^，HT可降低V20，但较3D-CRT提高了V10。Nguyen等^[23]^比较HT和3D-CRT技术在食管癌放疗中的应用，发现HT较3D-CRT技术更能降低MLD和最大脊髓剂量（*P*=0.004, *P* < 0.007）; Mavroidis^[24]^的研究中，HT较3D-CRT、IMRT不仅降低危及器官剂量，也降低了健侧肺剂量; 且在Kim等^[25]^用HT对31例NSCLC患者放疗的研究中，≥3级RP发生率为6.5%，患侧V5、V10、V15和健侧V5是RP发生的独立影响因素。说明HT在“低剂量大体积”的基础上更能限制危及器官剂量，保护健侧肺组织，降低RP发生。

Alexander等^[26]^用HT对中央型早期肺癌和肺转移灶进行立体定向放疗（stereotactic body radiotherapy, SBRT），处方剂量为：计划靶区（planning target volume, PTV）=7, 000 cGy/10 F，PTV边界至危及器官的距离为0.38 cm-0.85 cm，心脏、食管、脊髓的最高剂量分别为4, 900 cGy、2, 800 cGy和4, 400 cGy; 但Aibe等^[27]^也用TH对30例早期肺癌患者进行SBRT，其中2例出现5级RP，相关性分析中，只有GTV大小是5级RP发生的相关性因素（*P*=0.025）。因此，我们认为TH在中央型病灶放疗时对正常组织更有保护作用，但需控制GTV大小。

### 7 平衡参数控制下的调强放疗技术

目前，3D-CRT技术与IMRT技术中的谁更适用于肺癌的治疗尚无定论。以往在设计胸部放疗计划时，肺受量总是受到特别关注。随着RTOG0617结果的公布，心脏的受量和损伤受到关注^[28, 29]^，我们提出平衡参数控制下的调强放疗概念：既强调靶区剂量和靶区剂量适形度，又要平衡胸部就放疗涉及的各主要危及器官的受照剂量。这些危及器官包括肺、脊髓、心脏和食管（表 2）。

**2:** 肺癌处方剂量为60 Gy/30次时的剂量限制 Dose limitation at prescription of 60 Gy/30 F for lung cancer

Tumor or organ at risk	Dose limitation
Tumor^*^	PTV V95%≥97%
Lung^#^	Dmean≤17 Gy, V20≤34%, V5≤60%
Spinal cord^#^	Dmax≤50 Gy
Heart ^&^	Dmean≤35 Gy, V40≤80%, V60≤30%
Esophageal ^&^	Dmean≤34 Gy, Dmax≤105%
Vx: percentage volume of total lung exceeding x Gy; Dmean: mean dose; Dmax: max dose; ^*^: according to the ICRU report 83^[30]^; ^#^: according to research by Wang Jin^[11]^; ^&^ : according to results of RTOG 0617^[31]^.	

## 8 总结

IMRT技术和3D-CRT技术各具特点，从目前对肺癌的局部控制及损伤上看，IMRT技术和3D-CRT技术并无明显差异，但4D-CT/IMRT技术较3D-CRT技术相比可延长OS，更能保护正常肺组织，减轻肺损伤。h-IMRT技术可能降低肺剂量，但目前关于肺h-IMRT的研究普遍忽视了心脏和食管损伤，值得进一步研究。为此，我们根据ICRU83号报告、RTOG 0617结果及我们之前的研究结果，提出平衡参数控制下的剂量参数，以供参考。
